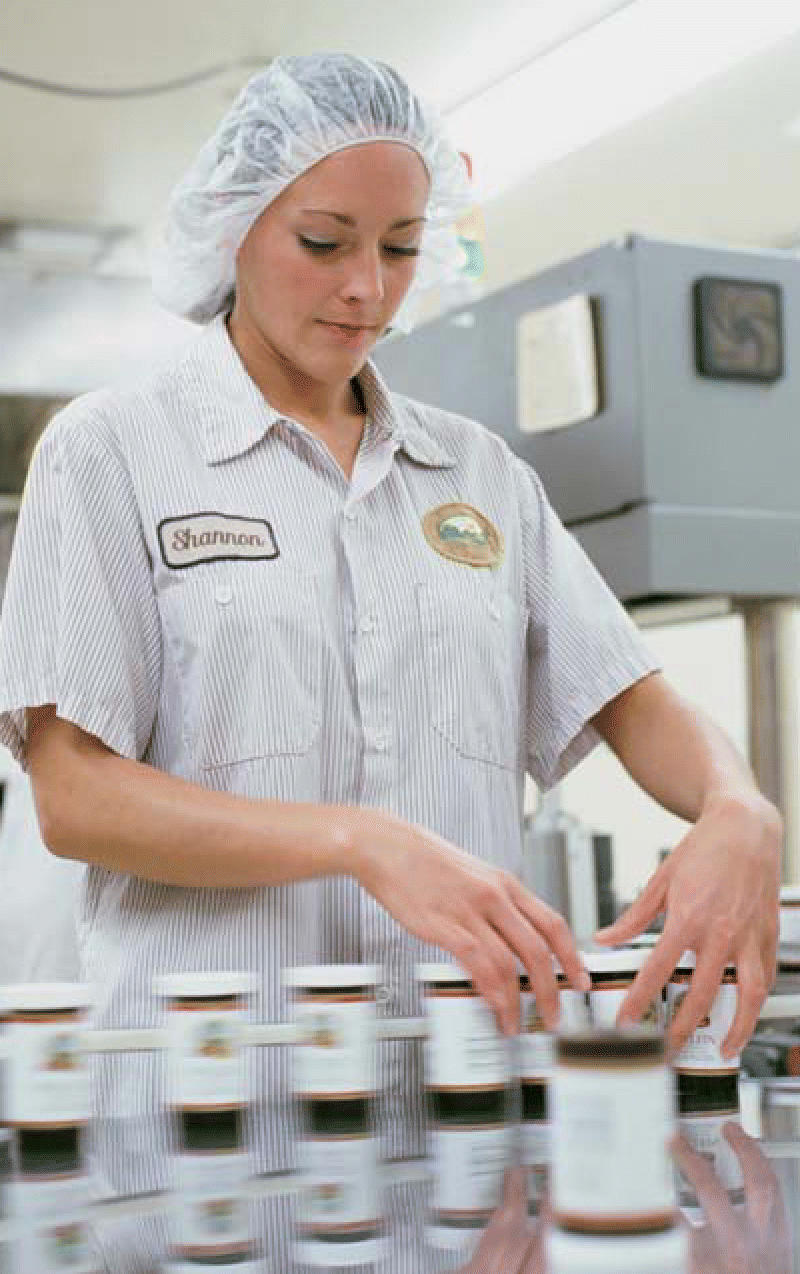# Headliners: Breast Cancer: Decreased Melatonin Production Linked to Light Exposure

**Published:** 2006-02

**Authors:** Tanya Tillett

Blask DE, Brainard GC, Dauchy RT, Hanifin JP, Davidson LK, Krause JA, et al. 2005. Cancer Res 65(23):11174–11184.

The incidence of breast cancer is up to fives times higher in women living in industrialized nations compared to those living in developing countries, and female night shift workers have particularly high rates of the disease. Given that Western nations have become “24-hour societies,” with more people awake around the clock, one hypothesis holds that nighttime exposure to artificial light suppresses the nocturnal production of melatonin. This hormone, produced by the pineal gland, helps regulate the body’s circadian rhythm and immune function, and also suppresses tumor growth. Now NIEHS grantees David E. Blask and George C. Brainard and their colleagues have confirmed that ocular exposure to bright artificial light at night inhibits the production of melatonin, which in turn may lead to an increased risk of developing breast cancer.

The researchers implanted human breast cancer cells into female laboratory mice, then transferred the malignant tumors that formed to female rats for continued development. They then collected blood from several healthy premenopausal volunteers under three different conditions: during the day, during the night following two hours of complete darkness, and during the night following 90 minutes of exposure to bright fluorescent light.

Next, they infused the collected blood directly through the developing tumors. Melatonin-rich blood collected following complete darkness slowed the growth of cancer tumors, while melatonin-depleted blood collected from volunteers exposed to both daylight and bright fluorescent light stimulated tumor growth.

The team also exposed tumor-bearing rats to varying intensities of light during the darkness phase of an alternating 12-hour light/12-hour dark cycle. They found that the extent to which melatonin production was suppressed depended on the magnitude of the light intensity that the rats were exposed to during the dark phase.

The authors say these results establish a role for the natural, nocturnal production of melatonin as a preventive agent in human disease. They also emphasize the risks of extensive exposure to bright artificial light at night, and point to the possibility that preserving the integrity of the circadian melatonin signal could help prevent breast cancer.

## Figures and Tables

**Figure:**